# Bacteria Detection: From Powerful SERS to Its Advanced Compatible Techniques

**DOI:** 10.1002/advs.202001739

**Published:** 2020-10-19

**Authors:** Xia Zhou, Ziwei Hu, Danting Yang, Shouxia Xie, Zhengjin Jiang, Reinhard Niessner, Christoph Haisch, Haibo Zhou, Pinghua Sun

**Affiliations:** ^1^ College of Pharmacy Jinan University Guangzhou Guangdong 510632 China; ^2^ Department of Oncology the First Affiliated Hospital of Jinan University Guangzhou Guangdong 510632 China; ^3^ Department of Preventative Medicine, Zhejiang Provincial Key Laboratory of Pathological and Physiological Technology Medical School of Ningbo University Ningbo Zhejiang 315211 China; ^4^ The Second Clinical Medical College (Shenzhen People's Hospital) Jinan University Shenzhen Guangdong 518020 China; ^5^ Institute of Hydrochemistry and Chair for Analytical Chemistry Technical University of Munich Marchioninistr. 17 Munich D‐81377 Germany

**Keywords:** bacteria detection, compatible techniques, label‐based, label‐free, SERS

## Abstract

The rapid, highly sensitive, and accurate detection of bacteria is the focus of various fields, especially food safety and public health. Surface‐enhanced Raman spectroscopy (SERS), with the advantages of being fast, sensitive, and nondestructive, can be used to directly obtain molecular fingerprint information, as well as for the on‐line qualitative analysis of multicomponent samples. It has therefore become an effective technique for bacterial detection. Within this progress report, advances in the detection of bacteria using SERS and other compatible techniques are discussed in order to summarize its development in recent years. First, the enhancement principle and mechanism of SERS technology are briefly overviewed. The second part is devoted to a label‐free strategy for the detection of bacterial cells and bacterial metabolites. In this section, important considerations that must be made to improve bacterial SERS signals are discussed. Then, the label‐based SERS strategy involves the design strategy of SERS tags, the immunomagnetic separation of SERS tags, and the capture of bacteria from solution and dye‐labeled SERS primers. In the third part, several novel SERS compatible technologies and applications in clinical and food safety are introduced. In the final part, the results achieved are summarized and future perspectives are proposed.

## Graphical abstract

This progress report focuses on the progress for bacterial detection by surface‐enhanced Raman spectroscopy (SERS) and other compatible techniques, and the contents mainly include the following parts: the enhancement principle and mechanism of SERS, the progress of label‐free strategy and label‐based strategy for SERS detection of bacteria, several novel developed SERS compatible technologies and the application in clinical and food safety.

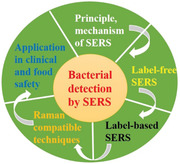

## 1 Introduction

Food‐borne diseases caused by microorganisms have become a serious problem in recent years. There are hundreds of millions of people in the world who suffer from diseases caused by food and water contaminated by pathogenic microorganisms each year. Reliable and fast bacterial detection is becoming an urgent need in public health assurance, medical diagnostics, and food safety fields. Currently, the most commonly used methods for microbial detection are mainly colorimetric method,^[^ ^1^, ^2^ ^]^ plate culture,^[^ ^3^ ^]^ flow cytometry,^[^ ^4^ ^]^ polymerase chain reaction (PCR),^[^ ^5^ ^]^ and enzyme‐linked immunosorbent assay (ELISA).^[^ ^6^ ^]^ These methods are reliable, highly specific, and selective, but also time‐consuming and laborious. They often take 2–3 d or longer for pathogen detection and identification, making them difficult to deal with sudden public health and safety incidents.

**Figure 1:**
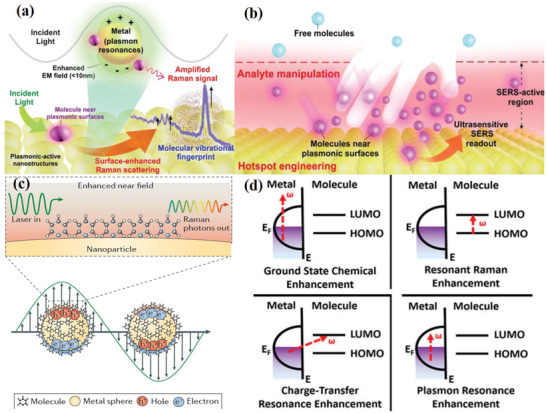
a) Schematic of LSPR effect of noble metal nanoparticles for SERS enhancement. b) Analyte techniques combine with “hot spots” to achieve ultrasensitive SERS readout. Reproduced with permission.^[^ ^8^ ^]^ Copyright 2019, Royal Society of Chemistry. c) Schematic of EM enhancement. Reproduced with permission.^[^ ^9^ ^]^ Copyright 2016, Springer Nature. d) Schematic of surface chemical enhancement. Reproduced with permission.^[^ ^10^ ^]^ Copyright 2015, American Chemical Society.

Surface‐enhanced Raman spectroscopy (SERS) is a promising spectroscopic analysis method for chemicals, disease markers, explosives, and microorganism detection.^[^ ^7^, ^8^, ^9^ ^]^ Owing to its high sensitivity, high resolution, fast data acquisition, and spectroscopic fingerprinting, SERS has attracted great attention for use in the detection and identification of pathogens in recent years. At the junctions between dimers and clusters in metal nanoparticles, aggregation produces strong electromagnetic field enhancement, which is known as “hot spots” effect. Based on the localized surface plasmon resonance (LSPR) (**Figure** **1**a) and “hot spots” effect (Figure 1b), the two recognized enhancement mechanisms are electromagnetic (EM) field enhancement (Figure 1c)^[^ ^9^ ^]^ and surface chemical enhancement (Figure 1d).^[^ ^10^ ^]^ When molecules are adsorbed on the surface of rough metals (e.g., Ag, Au, Cu) or metal nanoparticles (i.e., SERS substrate), the Raman scattering signals of the adsorbed molecules are strongly amplified up to 14 orders with highly enhanced SERS substrate, enabling the detection of a single molecule.^[^ ^11^ ^]^ With the development of biotechnology and nanomaterial technology, an increasing amount of research on bacterial detection using SERS technique has been reported, mainly focusing on the identification and classification of bacteria, microbial metabolic analysis, clinical microbe analysis, and microbiological analysis in water and food.

**Figure 2:**
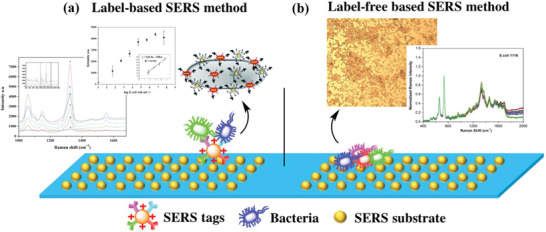
Schematic display of a) label‐based and b) label‐free method for SERS detection of bacteria. Reproduced with permission.^[^ ^12^ ^]^ Copyright 2017, Elsevier.

Thus far, the two principal methods for bacterial detection using SERS sensors are label‐free and label‐based strategies (**Figure** **2**).^[^ ^12^ ^]^ The label‐free method directly detects the original Raman signal of bacterial cells or bacterial metabolites based on the intrinsic vibrational fingerprint. This method is most commonly used for pathogen identification by processing data using statistical algorithms to analyze the differences in the Raman spectra. The label‐free strategy is simple and easy to conduct; however, the original Raman signal of bacteria is weak, making it a challenge to acquire highly reproducible spectral data. In label‐based bacterial detection, Raman signals are derived from the Raman reporter molecules of SERS tags, which produce distinct and ultrasensitive signals. The SERS tags bind with bacteria through specific recognition units; thus, bacteria can be indirectly detected by detecting the Raman signal of the reporters. Label‐based SERS detection is highly sensitive and reproducible; thus, multispecies detection can be achieved with different Raman reporter molecules. Compared with label‐free method, the label‐based strategy is highly sensitive and reproducible. However, it is also more complicated. It is worth noting that only the signature of reporters can be provided in a label‐based SERS strategy to achieve the further characterization, leading to the loss of intrinsic properties of bacteria.

In this review, advances in bacterial detection by SERS and other compatible techniques are discussed to summarize the development in recent years. The first part of this review is devoted to the label‐free strategy for the detection of bacterial cells and bacterial metabolites. In this section, we discuss important considerations that have to be made to improve the bacterial SERS signal, including the use of highly active SERS substrates, bacterial sample preparation, specific capture, and the concentration of bacteria in complex solutions. We then discuss the progress of the label‐based SERS strategy, including the design of SERS tags, the immunomagnetic separation of SERS tags, and the capture of bacteria from solution using dye‐labeled SERS primers. In the third part, several Raman‐compatible techniques are discussed. In the final part, we provide a summary of the key points and results achieved, and propose the steps that should be taken in the future.

## 2 Label‐Free Bacterial Detection Using SERS

For the label‐free method, the SERS spectra originate from whole bacterial cells or bacterial metabolites. In this section, we refer to the literatures and summarize the tentative assignments of typical SERS peaks of bacteria and bacterial metabolites (**Table** **1**).^[^ ^13^ ^]^

**Table 1:** Summary of typical SERS peaks of bacteria and bacterial metabolites

Peaks [cm^−1^]	Assignment^a)^	Originate
420	Skeletal modes CC	
550, 563		Carbohydrates
580	C—O—C	Glycosidic ring deformation
735	Glycosidic ring mode	Adenine
955	*υ*(CN)	
1040	CC ring breathing	
1460	*δ*(CH_2_)	Saturated lipids
1368	*υ*(COO—) and *δ*(C—H)	Proteins

^a)^ Approximate description of the modes (*υ*, stretch; *δ* and *γ*, bend).

### 2.1 Detection Signals Originating from Bacterial Cells

In this section, we discuss the progress of bacterial detection using Raman signals originating directly from bacterial cells and provide an overview of important factors that could be used for achieving a higher sensitivity and reproducibility of bacterial detection by label‐free SERS method.

#### 2.1.1 Enhanced Detection Sensitivity

With the development of nanometer synthesis technology, many kinds of metal nanomaterials have been prepared for use as SERS active substrates. The substrates for SERS detection are mainly gold (Au) and silver (Ag) nanoparticles (NPs). Usually, Ag NPs can provide significantly larger SERS enhancement than Au NPs. However, Au NPs are biocompatible, highly stable, and not easily oxidized. Generally, SERS substrates are mainly divided into two categories: monometallic and nanocomposite substrates. Moreover, the detected sensitivity of composite SERS substrate is higher than that of monometallic substrates.

##### Monometallic SERS Substrates

The synthesis methods, morphology, and surface properties of monometallic substrates directly affect the bacterial detection and analysis results. The simple mixing of bacteria and substrates usually results in the poor reproducibility of SERS spectra. To address this problem, the effective two synthesis methods of substrates are as follows:

##### In Situ Synthesis of Monometallic Substrates

Our team proposed in situ synthesis of Ag NPs on the bacterial cell wall for label‐free SERS detection.^[^ ^14^, ^15^, ^16^ ^]^ Similarly, the silver mirror reaction was used for the in situ synthesis of Ag NPs on the surface of bacteria by Alula et al.^[^ ^17^ ^]^ Gao et al. reported the aptamer in situ synthesis of Ag NPs to form well‐defined Staphylococcus aureus aptamer@Ag NPs.^[^ ^18^ ^]^ With this strategy, the enhancement and reproducibility of the Raman signals of bacteria are much greater than those of the simply mixed colloid‐bacterial method. Based on the discovery that the Zeta potential of the cell wall determines the bacterial intensity,^[^ ^14^ ^]^ our group subsequently demonstrated that Ag NPs coating bacterial structure (Bacteria@Ag NPs) can be utilized to calculate bacterial activity.^[^ ^19^ ^]^ Based on the structure of Bacteria@Ag NPs, our group analyzed *Escherichia coli* via antibody modification on the microarray platform to obtain stronger SERS enhancement.^[^ ^20^ ^]^ To achieve a higher sensitivity and reproducibility, our group detected *E. coli* by incubating bacteria with Ag NPs, and optimized the incubation conditions to obtain a higher SERS sensitivity and reproducibility compared with simple mixing.^[^ ^21^ ^]^

##### Green Synthesis of Monometallic Substrates

The need for energy saving and environmental protection has taken the green synthesis of nanoparticles in a new direction in recent years. Ag NPs are synthesized using leaf extracts of *Neolamarckia cadamba*; the resulting synthesized Ag NPs are very stable and are able to recognize bacteria of different compositions after being stored for three months.^[^ ^22^, ^23^ ^]^ Eggshells have been previously used as bio‐templates, as well as sputter coated with gold to fabricate stable and reproducible plasmonic substrates for the label‐free sensing and identification of *E. coli* and *Bacillus subtilis* bacteria.^[^ ^24^ ^]^ Intracellular Ag NPs can be used as SERS substrates for in vivo molecular probing. Wang et al. pointed out that the intensity of *E. coli* could be enhanced after dying with R6G.^[^ ^25^ ^]^ The surface of nanoparticles obtained by colloidal chemical synthesis is generally coated with a layer of nonbiocompatible substances, such as surfactants and anions, which reduces the accuracy of bacterial detection. To solve this problem, Kögler et al. prepared Au NPs and Au‐Si hybrid nanostructures for use in bacterial detection, and successfully identified *Listeria innocua* and *E. coli*. These laser‐synthesized nanomaterials have good biocompatibility and reduce the interference of bacterial detection.^[^ ^26^ ^]^

##### Different Morphologies of Monometallic Substrates

The morphology of the substrate is another factor that affects the sensitivity of bacterial detection. Silver nanomaterials have a variety of morphologies, such as nanoparticles, nanorods, and nanowires. Lemma et al. fabricated Ag NPs with high reproducibility that can directly detect the components of Gram‐negative and Gram‐positive bacteria, and as well as provide a SERS spectrum with a high signal‐to‐noise ratio.^[^ ^27^ ^]^ When Ag substrates of different morphologies are used in conjunction, a synergistically enhanced SERS signals can be obtained. Wang et al. reported a plasmonic nanoplate‐bacteria‐nanorod spherocrystals assay for the detection of different Gram‐positive and Gram‐negative bacteria. This new substrate not only enhances the multiple Raman scattering of the cells to be tested, but also distinguishes different bacterial species according to their SERS spectrum.^[^ ^28^ ^]^

##### Nanocomposite SERS Substrates

In recent years, with the development of nanosynthesis technology, multicomponent nanostructures have become a promising SERS substrates. There are an increasing number of reports on the use of bimetallic nanoparticles as SERS substrates, mainly because of their higher signal enhancement and better biocompatibility. Nanocomposite SERS substrates include metal–metal and metal–nonmetal forms.

##### Silver–Gold Bimetallic SERS Substrates

Bimetallic SERS substrates are comprised of an alloy and a core–shell structure. There are many reports regarding the use of silver–gold bimetallic SERS substrates for bacterial detection. For example, Sivanesan et al. prepared silver–gold bimetallic SERS substrates on the surface of electrochemically roughened nano‐silver using a simple electrodeposition method.^[^ ^29^ ^]^ The resulting substrate was very stable, and the SERS detection results for a variety of bacteria (*Salmonella enterica, E. coli, Bacillus megaterium*, and *Staphylococcus epidermidis*) showed that the Raman signal was significantly enhanced with good signal reproducibility.

##### Core–Shell Bimetallic Substrates

Qiu et al. successfully prepared a 3D Au–Ag core–shell structure with a controllable orientation. This super crystal structure, which is arranged vertically on the chip, can be used as a highly sensitive SERS substrate to detect and recognize Gram‐positive and Gram‐negative bacteria without markers.^[^ ^30^ ^]^ Jia et al. fabricated a nano‐assembly of Au@Ag nanorods, which is an excellent SERS active substrate. It is worth noting that this macro‐ordered 3D assembled structure perpendicular to the substrate could act as a homogeneous and highly active SERS substrate for the identification and detection of bacteria.^[^ ^31^ ^]^ This group later developed a long‐range ordered 3D Au/Ag core‐shell nanorod nano‐assembled structure for highly sensitive detection of *E. coli*.^[^ ^32^ ^]^ The core‐satellite structure can produce excellent SERS enhancement due to the “hot spots” generated efficiently between the plasma core and the satellite, which is widely used in optics.^[^ ^33^ ^]^ Qiu et al. fabricated a hybridized super substrate by combining the core‐satellite structure with the columnar array to provide high density “hot spots”. As a result, the SERS was enhanced to the maximum extent and the highly sensitive and direct detection of bacteria was realized.^[^ ^34^ ^]^ Huy et al. reported a novel nanocomposite composed of silver nanoparticles (Ag NPs) and manganese ferrite (MnFe_2_O_4_). This composite showed strong antibacterial activity against Gram‐negative bacteria and Klebsiella. Ag‐MnFe_2_O_4_ nanocomposite can also be used as SERS platform.^[^ ^35^ ^]^

##### Metal/Semiconductor Nanocomposite Substrates

Owing to their high surface‐to‐volume ratio, 3D metal/semiconductor nanostructures have attracted much attention as SERS substrates.^[^ ^36^, ^37^ ^]^ More recently, this hybrid nanostructure has been used to greatly increase the Raman scattering efficiency of bacteria. Prakash et al. used Ag–TiO_2_ nanocomposites as SERS substrates.^[^ ^38^ ^]^ Ko et al. developed a multifunctional Ag/ZnO/RGO nanocomposite by combining the photocatalytic properties of ZnO, a high surface‐to‐volume ratio, and the near‐ infrared photothermal conversion of RGO, bactericidal properties, and SERS properties of Ag nanoparticles. The composite can be used for the detection and inactivation of many kinds of bacteria.^[^ ^39^ ^]^

##### Metal–Polymer Composite Substrates

It has been reported that a hybrid surface composed of metal nanoparticles and polymers may be helpful for generating more significant bacterial SERS signals.^[^ ^40^ ^]^ Bhunia et al. prepared a flexible, composite film with C‐dots and Ag NPs coating. When bacterial samples were deposited on the polymer film, they showed unique SERS characteristics.^[^ ^41^ ^]^ A unique 3D SERS substrate was prepared by combining multilayer Ag NPs with a polymer brush, which can be used for the efficient detection of *S. aureus*. The limit of detection (LOD) was down to 8 CFU mL^−1^ based on this 3D SERS substrates.^[^ ^42^ ^]^ Kumar et al. also fabricated silver nanorods (Ag NRs) array poly(dimethylsiloxane) (PDMS) substrate. The SERS enhancement of *Pseudomonas aeruginosa* was realized by producing buckles on the PDMS substrate of Ag NRs array, resulting in the formation of high density “hot spots” in the Ag NRs array. The Ag NR‐PDMS buckling system has good encapsulation performance, which increases the net effective contact area between the bacteria and metal surface.^[^ ^43^ ^]^ Cao et al. prepared large‐area planar thin layers with pores covered with Au NPs for used as sensitive SERS substrates in bacterial sensing.^[^ ^44^ ^]^ This approach produced uniform and repeatable large‐ area surfaces, and improved the sensitivity of the Raman spectrum.

##### Metal–Nonmetallic Nanocomposite SERS Substrates

Several studies have attempted to synthesize functional nanomaterials composed of metal and nonmental for bacterial detection. Hardiansyah et al. prepared a unique hybrid structure composed of Au NPs, silicon dioxide, and iron platinum, which greatly enhanced the rapid identification of *S. aureus* by SERS.^[^ ^45^ ^]^ Hartley et al. used black silicon‐based substrates to insert the bacterial peak on the nanoscale SERS peak to obtain the spectrum of the internal composition information of bacteria. Therefore, it can be used for insertion and SERS identification of bacteria.^[^ ^46^ ^]^ Li et al. designed a structure for the synthesis of Ag NPs on rGO sheets. Owing to the synergistic effect of Ag NPs and rGO, the LOD of *E. coli* was reduced to 10^5^ CFU mL^−1^.^[^ ^47^ ^]^

##### Polymer Fiber Probe

**Figure 3:**
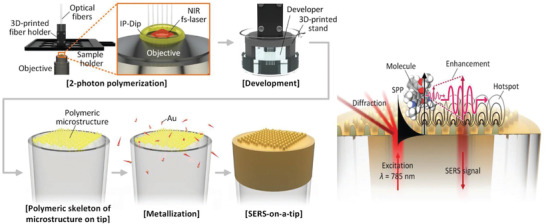
Schematic diagram, preparation process, and working principle of SERS‐on‐a‐tip probe. Reproduced with permission.^[^ ^50^ ^]^ Copyright 2020, Wiley.

Because polymer fiber has some unique properties, such as good flexibility, high compactness, and strong in situ remote sensing ability, the polymer fiber structure composed of embedded or modified Au/Ag NPs is a good flexible SERS detection platform. Based on high‐performance remote sensing of femtosecond laser ablation and photochemical reduction deposition of Ag NPs, Yin et al. reported a U‐shaped fiber SERS probe for biochemical analysis.^[^ ^48^ ^]^ Tian et al. used gold nanorods (Au NRs) as plasmonic nanostructures to fabricate 3D SERS substrates based on a biosynthesized bacterial nanocellulose (BNC) fiber. This 3D porous BNC structure is conducive to the uniform and dense adsorption of bacteria on the surface and sub‐surface of the plasma nanostructure, and provides an ideal SERS platform for the collection, detection and identification of bacteria.^[^ ^49^ ^]^ Yang et al. used a two‐step protocol to present a novel fiber‐optic SERS probe based on off‐the‐shelf components and materials.^[^ ^50^ ^]^ This fiber‐optic SERS probe was used to detect live, unlabeled bacteria with higher controllability and repeatability (**Figure** **3**).

#### 2.1.2 Capture and Immobilization of Bacteria

Effectively capturing and immobilizing bacteria is of great importance to improve the selectivity and specificity in bacterial detection, especially in complex real‐world bacterial samples. In this section, we will discuss efforts to capture and immobilize bacteria from samples prior to detection by SERS.

##### Nonspecific Capture

**Figure 4:**
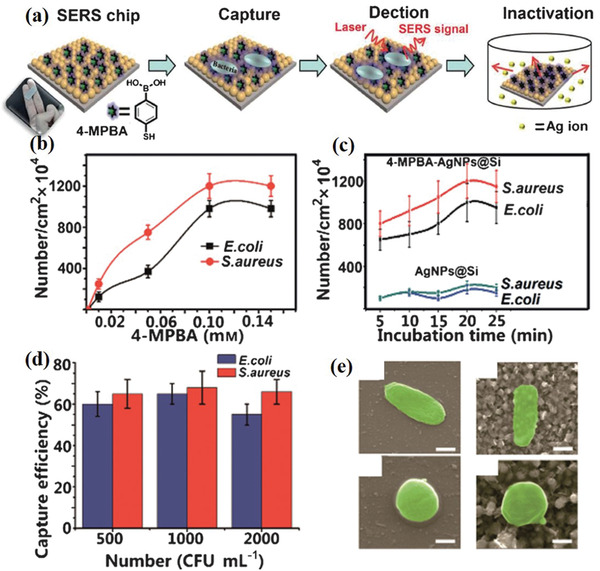
a) Schematic illustration of the function of 4‐MPBA in bacteria capture and SERS detection. The number of bacteria at different b) concentrations, c) incubation times. d) Bacterial‐capture efficiency at low bacterial concentrations. e) SEM images of a single bacterium attached on silicon wafer and the SERS chip (scale bars: 500 nm). Reproduced with permission.^[^ ^53^ ^]^ Copyright 2015, Wiley.

The receptor‐free immobilization of bacteria is usually nonspecific. There are two methods for capturing bacteria. The first is based on electrostatic forces. Since the nuclei of lipopolysaccharide molecules of bacteria are phosphate and carboxylate, the surface of the bacterial membrane is negatively charged. In a previous study, a positively charged nanocomposite Au/graphene‐poly (diallyldimethylammonium chloride) nanocomposite SERS substrate was used to easily capture the negative charge of *S. aureus* for SERS detection.^[^ ^51^ ^]^ In another study, *Salmonella typhimurium* was immobilized on the positively charged poly(L‐lysine)‐coated 3D plasmonic nanopillar arrays through electrostatic interactions.^[^ ^52^ ^]^ With the help of 4‐mercaptophenylboronic acid (4‐MPBA), another method can be used to capture bacteria. The 4‐MPBA molecule contains three functional groups: a thiol group for conjugation with Ag NPs^[^ ^53^ ^]^ or Ag dendrite substrate,^[^ ^54^ ^]^ a boronic acid group that binds to peptidoglycan from the cell wall of bacteria reversibly, and thus can capture various kinds of bacteria, and a benzene ring that significantly amplifies the SERS signals of the captured bacteria (**Figure** **4**). Compared to electrostatic interactions, using 4‐MPBA as a bacterial capture agent has a better specificity.

##### Specific Capture of Bacteria

Specific capture probes are often used to improve the detection selectivity and capture of the target bacteria from complex samples. In this section, we discuss commonly used bacteria‐specific capture units, including antibodies, antibiotics, aptamers, bacteriophages, and antimicrobial peptides.

Antibodies are the most popular capture elements for SERS detection because of their high affinity and specificity for bacteria. Some studies have successfully employed antibody‐conjugated nanoparticles to achieve the specific capture of bacteria from various complex samples.^[^ ^55^, ^56^, ^57^ ^]^ In addition to antibodies, antibiotics that interact specifically with bacterial cell walls can also be used as capture probes for bacteria. Vancomycin is one of the most commonly used antibiotics for bacterial capture in SERS detection. It has been proven that an antibiotic vancomycin coating can significantly increase the adhesion of bacteria on nano‐interfaces and improve the SERS response of bacteria.^[^ ^58^ ^]^ Wu et al. reported that the intrinsic SERS spectra of vancomycin‐coated Ag nanorods can be used to analyze 27 different strains from 12 bacterial species.^[^ ^59^ ^]^ Although antibodies have a good specificity and selectivity, the stability of antibodies is low, which is not conducive to their long‐term preservation. Moreover, the price of antibodies is usually high. In recent years, aptamers have become a popular alternative to antibodies. An aptamer‐binding label‐free SERS detection method has been established on DNA aptamer‐modified Ag NRs substrates. The specificity of *S. typhimurium* was improved with negative control bacteria.^[^ ^60^ ^]^ Based on the hybridization of the DNA aptamer and the complementary DNA, an SERS‐based aptasensor for *S. typhimurium* detection can be constructed.^[^ ^61^ ^]^

A bacteriophage (phage) is a virus that infects bacteria. Since bacteriophages have specificity for bacterial hosts, they can be used as specific recognition elements for bacteria in SERS detection. The Tbilisi phage is the main representative of Brucella‐specific SERS detection.^[^ ^62^ ^]^ Even at low concentrations, good bacterial capture efficiency and fast antibacterial rate are the main advantages of antimicrobial peptides over other capture methods.^[^ ^63^ ^]^ However, bacterial detection using antimicrobial peptides as capture and recognition elements is based on electrochemical method,^[^ ^63^, ^64^, ^65^ ^]^ and fluorescence method,^[^ ^66^, ^67^ ^]^ antimicrobial peptides applied in SERS detection have rarely been reported. The advantages and disadvantages of these capture and recognition methods are summarized in **Table** **2**, with the aim of providing readers with a reference when selecting the appropriate bacterial capture method in order to optimize SERS detection.

**Table 2:** Summary of advantages and disadvantages of these capture and recognition methods

Recognition elements	Advantages	Disadvantages
Electrostatic interaction		Lack of specificity
4‐MPBA	Stable, low cost	Poor specificity
Antibody	High affinity and specificity	Unstable, expensive
Antibiotic	Good specificity	Less binding sites
Aptamer	High affinity and specificity, chemically stable, easy to be modified	Very few types of aptamers have been screened for bacteria recognition
Antimicrobial peptide	Stable, low cost, high affinity and specificity, more binding sites	Specificity is slightly lower than antibodies
Phage	Stable, high specificity for the host bacteria	

#### 2.1.3 Concentration of Bacteria

In the practical applications of bacterial detection and identification, the bacterial concentration of samples from various sources is usually very low. One common way to increase the concentration of bacteria is through culturing. However, this is time consuming. Therefore, there is currently an urgent need to develop new bacterial concentration methods with which to improve the accuracy and sensitivity of bacterial detection. Alternative methods include filtration, magnetically assisted separation and enrichment.

##### Filtration

The following advances have been made with regards to SERS active nanoparticles and filtration: 1) an enhanced Raman signal amplitude based on the resonance effect; 2) an improved reproducibility; 3) improved ability to collect targets from low‐ concentration samples; 4) uniform distribution of nanoparticles and bacteria onto the surface of SERS substrates.

**Figure 5:**
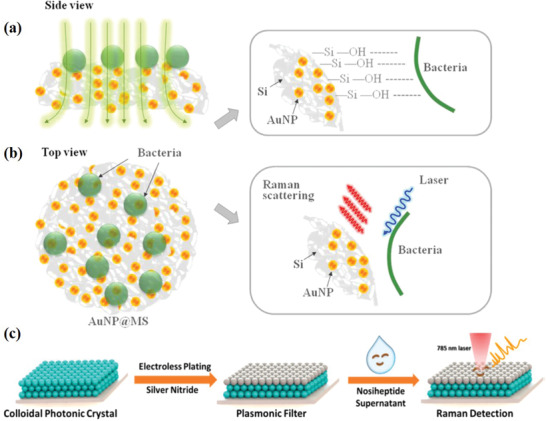
a) The hydrogen bond between bacteria and silanol group is directly attached to the substrate surface. b) SERS enhancement produced by Au NPs is used for bacterial detection. Reproduced with permission.^[^ ^70^ ^]^ Copyright 2014, Elsevier. c) Schematic of plasmonic filter design and Streptomyces spores discrimination. Reproduced with permission.^[^ ^72^ ^]^ Copyright 2017, American Chemical Society.

In some cases, Ag or Au NPs have been used as filters. In this way, bacteria can be filtered and evenly distributed on the surface of SERS substrates.^[^ ^68^, ^69^ ^]^ Many similar studies on filter‐like substrates for bacterial detection can be found in the literature. For example, Lin et al. prepared Au NPs dispersed in mesoporous silica (Au NPs@MS) substrates. The substrates improve the filtration of *S. aureus* in water samples. The target cells were concentrated on the Au NPs@MS substrates within several seconds, and an improved reproducibility of the SERS spectra was obtained (**Figure** **5**a,b).^[^ ^70^ ^]^ Recently, this group fabricated Ag NPs‐decorated SERS filters via silver‐mirror reaction.^[^ ^71^ ^]^ They demonstrated that this filter‐like substrate produced significant intensity and clearer peaks, thus producing strong SERS signals with good bacterial discrimination. Wang et al. designed and prepared a self‐assembled Ag NPs‐coated photonic crystal to prepare a new type of plasma filter. Thanks to the even distribution of “hot spots” on the surface of the filter and the high density, the filter was used to successfully filter, capture, and identify *Streptomyces* spores via SERS (Figure 5c).^[^ ^72^ ^]^

The filtration procedures and SERS can be combined for the detection of low concentrations of bacteria.^[^ ^73^ ^]^ Some research groups have integrated SERS with a universal sample preparation process or unique devices to achieve rapid, sensitive, and real‐time bacterial detection from various samples. Simple extraction procedures, such as liquid−liquid extraction, to concentrate targets combined with SERS‐based detection methods can be used for the quantification of metabolites (e.g., p‐coumaric acid).^[^ ^74^ ^]^ From a clinical perspective, many challenges still lie in the way of direct detection of infectious bacteria in blood, including low bacteria concentrations (<10 CFU mL^−1^), as well as large number of blood cells and high content of protein interference impurities per mL of blood. A SERS‐platform has been previously used to separate bacteria from human blood. Boardman et al. designed a device to filter 10 mL of whole blood and produce rich live bacteria for SERS analysis.^[^ ^75^ ^]^ A nonwoven polymer was used as the filter membrane to separate the bacteria from the blood, the former of which were fixed on the surface of the membrane during measurement.^[^ ^76^ ^]^

##### Magnetically Assisted Separation and Enrichment

Bacteria can be fixed on the surface of Fe_3_O_4_ magnetic nanoparticles to combine with the object to be tested, allowing the target to be easily separated under the effect of an external magnetic field, according to its magnetic characteristics. At present, magnetic separation is widely used for the enrichment and separation of heavy metal ions, environmental toxins, and biologically active molecules, such as cells, proteins, DNA, viruses, and bacteria.^[^ ^77^ ^]^ Recently, magnetic nanoparticles and commonly used metal nanoparticles were combined to form Ag/Au‐doped magnetic nanocomposite nanoparticles, which were not only able to retain the excellent properties of Ag/Au nanoparticles, but also had the magnetic properties of magnetic nanoparticles, showing great potential in clinical applications. Among them, magnetic nanocomposites with core–shell structures are one of the most widely studied and used magnetic composites. It is worth noting that core–shell popcorn‐like NPs can be used as “photoconductive nanoheaters” to destroy multidrug‐resistant bacteria (MDRB) *Salmonella* DT104 at high temperatures using near‐infrared light. Fargasova et al. developed magnetic nanoparticles (MNPs) modified by streptavidin (Strep). The surface of the MNPs@strep was functionalized with an appropriate antibody, and then coated with Ag NPs for the SERS detection of bacteria.^[^ ^78^ ^]^ Several hybrid nanostructures have been synthesized using multiple nanoparticles. For instance, Hardiansyah et al. developed a new structure consisting of iron platinum (FePt), silica (SiO_2_), and Au NPs (FePt@SiO_2_‐Au), which displayed magnetic separation capability.^[^ ^45^ ^]^

### 2.2 Detection Signals Originating from Bacterial Metabolites

In contrast to bacterial cells, spores are dormant and have a high resistance to drying and extreme temperatures. The high concentration of dipicolinic acid (DPA) in bacterial spores is important for the preservation of spore DNA.^[^ ^79^, ^80^ ^]^ Therefore, DPA detection is currently an active area of research.^[^ ^81^ ^]^ In order to reduce the total mass of DPA, mesoscopic droplet operations have been performed on samples. The advantage of this type of sampling is that the sample volume can be significantly reduced to ensure Raman detection, thus reducing the amount of required sample. Therefore, in this case, the amount of DPA required for detection can be reduced by two orders of magnitude.^[^ ^82^ ^]^ These involve the direct detection of DPA with SERS. Recently, Bai et al. reported an ion‐assisted DPA method based on DPA–Hg^2+^ complex for the ultrasensitive SERS detection of DPA with LOD of 0.01 ppb. This method allows for the full capture of DPA, as well as non‐interference detection in complex environments. Compared with the existing DPA determination method, the accuracy and reliability of the determination are greatly improved.^[^ ^83^ ^]^

**Figure 6:**
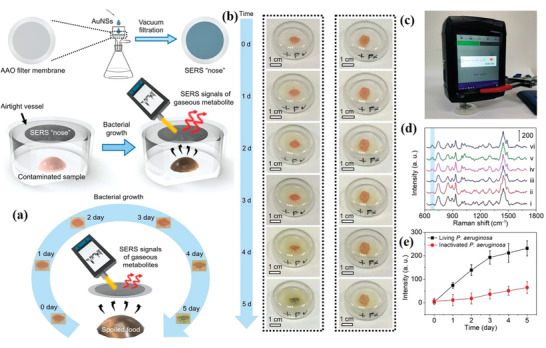
a) Schematic illustration to fabricate SERS “Nose” and monitor SERS signals of gaseous metabolites. b) Optical photos of pork inoculated with living and inactivated *P. aeruginosa* at different culture time (0–5 d). c) A hand‐held Raman spectrometer for SERS detection. d) Typical SERS spectra for the culture times of 0–5 d (i−vi), respectively. e) Plot of SERS intensity at 680 cm^−1^ versus culture time. Reproduced with permission.^[^ ^86^ ^]^ Copyright 2020, American Chemical Society.

Using short peptides to bind to the cholera toxin as a recognition element, the selective detection of cholera toxin based on SERS was achieved, with the LOD of 3.51 pg mL^−1^. The sensitivity and specificity were much greater than those previously reported.^[^ ^84^ ^]^ Specific antigen fraction 1 (F1) is a protein marker of *Yersinia pestis*. A LOD of 59.6 pg mL^−1^ of F1 antigen was achieved using an integrated SERS‐based microdroplet platform.^[^ ^85^ ^]^ Recently, Ju et al. designed and produced a SERS “nose” to quickly monitor the gas metabolites of bacteria. The gold nanostars (Au NSs) were loaded on the plane filter support with vacuum filtration. Their intensive accumulation leads to high sensitivity of SERS signal, especially to the bacterial gaseous metabolites, thus was called as SERS “nose”. The “nose” had a good reproducibility when it used for the quantitative detection of gas targets by SERS (**Figure** **6**).^[^ ^86^ ^]^

## 3 Label‐Based Bacterial Detection Using SERS

### 3.1 SERS Tags and Raman Reporter Molecules

SERS labels are optical nanoprobes that combine noble metal nanoparticles with specific organic Raman signal molecules. The typical nanoprobes structure is mainly composed of plasma metal nanoparticles, organic Raman labeled molecules, a protective layer and target recognition molecules.^[^ ^87^ ^]^ The metal nanostructure acts as a rigid substrate and enhances the Raman signal by enhancing localized electromagnetic field. By changing the morphology, composition, size, and structure of metallic nanoparticles, the performance of SERS tags can be changed, thus expanding its application. Conjugating SERS‐active nanosubstrates to Raman signal molecules with a characteristic Raman signal is an important step in preparing SERS tags. SERS tags produce strong characteristic Raman signals by adsorbing Raman signal molecules. The main types of Raman signal generation are thio‐small molecular substances, nitrogen cations and sulfur dyes. Nile Blue A (NBA), 4‐aminothiophenol (4‐ATP), crystal violet (CV), 4‐mercaptobenzoic acid (4‐MBA), and rhodamine 6G (R6G) are mostly used as Raman reporters. The characteristic peaks of typical Raman signal molecules by the label‐based SERS method are shown in **Table** **3**. The traditional Raman signal molecules show multiple spectral bands in the fingerprint region, which usually overlap with the spectral bands of bio‐macromolecules, whereby it is difficult to separate them. To solve this problem, some background‐free Raman signals, such as alkyne (C≡C) and nitrile (C≡N), have been reported.^[^ ^88^, ^89^, ^90^ ^]^ These new Raman signal molecules show a single peak in the Raman silent region (1800–2800 cm^−1^), which can mitigate spectral confusion and background interference, and is a good choice for the analysis of biological samples. The structure of bare nanoparticles with Raman reporter molecules lacks stability, and the signal may be affected by the surrounding medium. Therefore, it is necessary to encapsulate a protective layer outside this structure to enhance its biocompatibility and prevent its aggregation. Therefore, many kinds of surface coating materials (e.g., silica, liposomes, polymers, and biomolecules) have been used to improve the stability of SERS signals. Finally, recognition elements, such as antibodies and aptamers, are introduced for the identification of targeted bacteria.

**Table 3:** Typical types of Raman signal molecules for SERS detection of bacteria

Type	Raman signal molecules	Linking mode	Characteristic peaks [cm^−1^]
Nitrogen‐containing cationic dye	Rhodamine 6G (R6G)	N—Au (Ag)	1511
	Cyanine dye 3 (Cy3)		1590
	QSY21 dye	Electrostatic interaction	1496
	Ethyl violet (EV)		800, 1620
Sulfur‐containing dyes	Malachite green isothiocyanate (MGITC)		1170, 1616
	DTDCI		1132,1240, 1578
	Tetramethyl rhodamine isothiocyanate (TRITC)		1217,1357,1649
Thio‐small molecules	4‐mercaptobenzoic acid (4‐MBA)	S—Au (Ag)	1587, 1080
	5,5‐dithiobis‐2‐nitrobenzoic acid (DTNB)	S—Au (Ag)	1331
	4‐aminothiophenol (4‐ATP)	S−Au (Ag)	1079, 1590

### 3.2 Design Strategy of Label‐Based SERS Nanobiosensor for Bacterial Detection

#### 3.2.1 SERS Tags for Bacterial Detection

**Figure 7:**
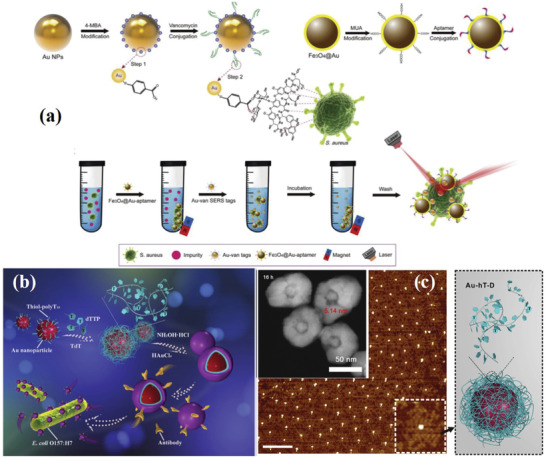
a) Schematic illustration of the synthesis of Au‐Van SERS tags and the procedure for *S. aureus* detection via the dual‐recognition SERS biosensor. Reproduced with permission.^[^ ^96^ ^]^ Copyright 2019, Elsevier. b) Schematic illustration of core–shell nanostructure combined with TdT‐catalyzed DNA for the detection of *E.coli* O157: H7 cells. c) AFM and TEM image of the composite structure. Reproduced with permission.^[^ ^104^ ^]^ Copyright 2019, Elsevier.

To improve the performance of SERS tags in bacterial detection, various metal substrates have been employed for the signal enhancement, such as Au and Ag NPs,^[^ ^91^ ^]^ Au@Ag nanorods,^[^ ^92^ ^]^ SiO_2_@Au core/shell NPs,^[^ ^93^ ^]^ oxide@Au nanoovals,^[^ ^94^ ^]^ and Ta@Ag array.^[^ ^95^ ^]^ The enhancement factor (EF) is far greater than 10^10^, and can be used for the SERS detection of a single molecule. Therefore, the aggregation of metal nanoparticles has been widely developed as a highly sensitive SERS substrate for labeling. In order to achieve the highly selective detection of single bacteria, a special structure of the SERS nanoprobe, namely specific antibody binding nanoaggregate embedded beads (NAEBs), was introduced into the SERS labeling method. NAEBs are usually stable colloids, which are obtained by controlling the aggregation of Au NPs coated with Raman signal molecules on the surface of protective shell (**Figure** **7**a). After NPs aggregation, high‐density plasma “hot spots” are generated at the junction of Au NPs to obtain strong SERS signal of Raman signal molecules.^[^ ^96^ ^]^ The mimetic bacteria were detected by their SERS signal after binding antibody‐conjugated NAEBs to antigen‐functionalized polystyrene microspheres.

In order to establish the SERS tags for the specific detection of target bacteria, it is necessary to modify antibodies, aptamers or small molecule ligands. Antibodies and aptamers are mostly used. Aptamers with high affinity and selectivity are gradually becoming the substitutes for antibodies for the sensitive and rapid detection of bacteria. Duan et al. reported a lable‐based aptasensor SERS approach for quantitative detection of *S. typhimurium* or *Vibrio parahaemolyticus*.^[^ ^93^, ^97^ ^]^ Moreover, other recognition elements have also been introduced for the identification of targeted bacteria. For example, Tay et al. reported that the detection of *Salmonella* cells can be reduced to the limit of a single cell by combining with the NAEBs of *Salmonella‐*specific tail spike protein isolated from P22 phage.^[^ ^98^ ^]^ T‐4 bacteriophages combined with nanosilver membrane immobilization technology were used for *E. coli* capture.^[^ ^99^ ^]^ Monosialoganglioside is a natural glycolipid molecule that has been shown to act as a high‐affinity and specific ligand for cholera toxin secreted by *Vibrio cholera*.^[^ ^100^ ^]^ By fabricating various SERS tags with different Raman reporter molecules and different specific recognition elements, the simultaneous detection of multiple pathogens can be achieved. Based on this strategy, the simultaneous detection of *S. aureus* and *S. typhimurium* has been demonstrated.^[^ ^101^ ^]^ The LOD of *S. typhimurium* is 15 CFU mL^−1^, and that of *S. aureus* is 35 CFU mL^−1^. Wang et al. detected *Salmonella* enterica serovar *Typhimurium* and *Staphylococcus* simultaneously, with the lowest detection concentration in spinach solution (10^3^ CFU mL^−1^). With different coded particles used for each targeted pathogen, along with their corresponding labeling molecule, four types of pathogens from the blood of infected patient were identified.^[^ ^102^ ^]^ Ma et al. developed a simple, sensitive, rapid, and highly selective biosensor for *Salmonella*. Among them, 4‐mercaptobenzoic acid (4‐MBA) was selected as the Raman signal molecules, and the aptamer of *Salmonella* thio‐formate was used as a SERS nanoprobe to functionalize spiny gold nanoparticles (SGNPs). The biotin aptamer can be fixed on a micro‐titration plate to realize the specific recognition and capture of the fungus. According to the relationship between the SERS intensity of 4‐MBA peak at 1586 cm^−1^ and the concentration of bacteria within a certain range, the LOD was successfully down to 4 CFU mL^−1^.^[^ ^103^ ^]^ When the target is special bacterial gene, the hairpin DNA structure modified by 3′‐ thiol and 5′‐biotin was covered on Au@Ag NRs as a SERS marker for the detection of specific gene fragments of *Bacillus thuringiensis* (BT). In the presence of the target, the ring hybridizes with the target, opens the hairpin, and is captured by the magnetic nanoparticles modified by streptavidin, which reduces the suspended NRs and leads to the change in SERS intensity. Based on this sensor, the LOD of 0.14 × 10^−12^ m for Bt transgene fragment was achieved.^[^ ^92^ ^]^ Yan et al. combined core–shell nanostructures with deoxynucleotidyl transferase (TdT)‐catalyzed DNA for the detection of *E. coli* O157: H7 cells. The obtained elongated products, mononucleotide long single strand DNA (hn‐D), can induce tunable nanogaps and obtain uniform signal enhancement. In addition, the specific Raman imaging of *E. coli* O157: H7 has a good performance, with a high detection sensitivity (up to 2 CFU mL^−1^), and a recovery rate in actual food samples of 98.1–105.2% (Figure 7b,c).^[^ ^104^ ^]^

#### 3.2.2 Immunomagnetic Separation of SERS Tags and Captured Bacteria from Solution

**Figure 8:**
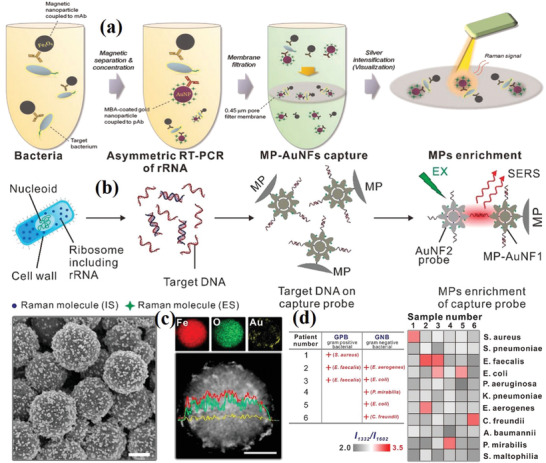
a) Schematic diagram of membrane filtration combined SERS for the detection of *E. coli*. Reproduced with permission.^[^ ^107^ ^]^ Copyright 2015, Elsevier. b) A self‐calibrating SERS system for phenotype detection of bacteria. c) SEM, HAADF‐STEM images and EDS elemental mapping of the prepared Au NFs. Scale bar: 400 nm. d) Heat map of obtained *I*_1332_/*I*_1602_ for practical sample analysis. Reproduced with permission.^[^ ^109^ ^]^ Copyright 2020, American Chemical Society.

The detection of pathogenic microorganisms in complex biological samples is difficult because of the complexity of low pathogen concentrations, as well as the presence of matrices in biological samples. Immunomagnetic beads separation (IMS) technology has become a promising way to overcome these difficulties. A highly sensitive and selective sandwich immune SERS analysis method has been established based on the magnetic properties of nanoparticles. The sandwich immunoassay is comprised of a capture probe and a signal probe. The target bacteria were collected and concentrated via the immunomagnetic separation of specific recognition elements combined with magnetic nanoparticles instead of simple centrifugation to avoid the agglomeration of sample particles. Then, the combination of SERS tags and recognition elements as SERS signal probes were reacted with the pathogen‐coated magnetic nanoparticles through an immune response. Finally, pathogen‐coated SERS probe was subjected to Raman analysis. Wang et al. designed a magnetically assisted SERS bioassay for the single‐cell detection of *S. aureus*.^[^ ^105^, ^106^ ^]^ The Ag‐coated magnetic nanoparticles (Ag MNPs) were designed to achieve a good antibacterial effect (up to 75%) . The LOD for *S. aureus* was 10 cells mL^−1^. Cho et al. used immunomagnetic separation and membrane filtration technology to separate, capture, and concentrate the target bacteria encapsulated by nanoparticles from beef samples to effectively remove the interference signals of unbound components and background bacteria in the process of SERS detection (**Figure** **8**a).^[^ ^107^ ^]^ Kearns et al. developed a new type of biosensor based on magnetic bead separation and SERS technology for the separation and detection of various pathogens. The basic principle of this new analytical model is to use functional magnetic nanoparticles to capture and isolate bacteria from the sample matrix, and then use SERS active nanoparticles modified by strain‐specific antibodies to selectively detect bacterial pathogens. Therefore, this method, which combines “magnetic plugs” to capture samples and specific antibody modification, allows for the simple and rapid optical detection of a variety of bacteria. The experimental results show that this method can successfully isolate and detect *E. coli, S. typhimurium*, and *S. aureus*.^[^ ^108^ ^]^ Li et al. designed a self‐calibration SERS sensor based on the specific and sequence DNA hybridization assembly between gold nanoflower (Au NFs) with an internal standard, and obtained a high sensitivity and quantitative analysis of biomolecules. This stable and reliable SERS sensor can distinguish different kinds of bacteria and realize a high sensitivity detection of a single bacteria to be used for the high repeatability detection of low concentrations of pathogen in actual samples (Figure 8b–d).^[^ ^109^ ^]^

## 4 Raman‐Compatible Techniques for the Detection of Bacteria

### 4.1 Mapping Technique

**Figure 9:**
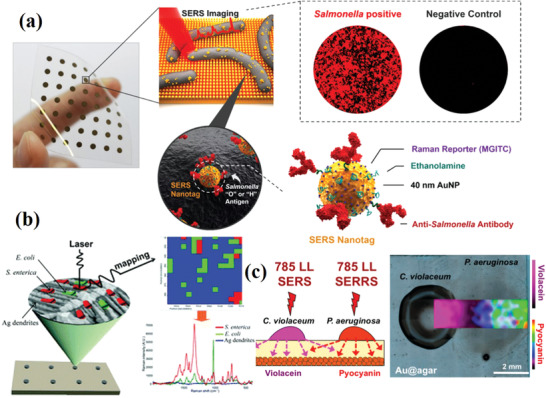
a) Bacterial pathogens were immobilized on a 3D plasmonic nanopillar arrays. Then, the surfaces of bacteria were labeled with SERS tags, and further quantitative analysis for SERS detection through the collection of Raman mapping for statistical analysis. Reproduced with permission.^[^ ^52^ ^]^ Copyright 2018, American Chemical Society. b) Schematic diagram of label‐free mapping of a single bacterial cells using SERS. Reproduced with permission.^[^ ^111^ ^]^ Copyright 2016, Royal Society of Chemistry. c) Schematic diagram of imaging bacterial interspecies chemical interactions by SERS. Reproduced with permission.^[^ ^79^ ^]^ Copyright 2017, American Chemical Society.

Raman mapping is an effective method to study and quantify the distribution and peak analysis of different components and their specific regions. In each pixel of the defined area, hundreds of spectra can be automatically collected, then analyzed and synthesized according to the intensity of the specified peak, ultimately forming a color image. Raman mapping has been widely used in many methods of bacterial cell detection based on SERS technology. The in situ SERS spatiotemporal mapping of the bacterial community has been proven to be an effective tool for comprehensively characterizing the cell–cell relationship in the colony.^[^ ^110^ ^]^ SERS mapping can also be used for spatial distribution, visualizing expression, and microbial metabolites in the coculture (**Figure** **9**).^[^ ^79^ ^]^

SERS mapping images can be used for the quantitative analysis of bacteria. In a typical SERS mapping process, a defined mapping area is first identified. Then, mapping data are collected with a selected step size. After point‐by‐point scanning of the mapping area, hundreds of data points can be obtained for the bacterial sample. The SERS mapping image is constructed based on the intensity of the selected characteristic peak. With various concentrations of bacteria, the mapping images contain different numbers of bright spots. By scanning through bacterial cells on the surface of the SERS substrate, single bacterial cell detection was achieved with LOD of 10^4^ CFU mL^−1^, and the detection limit 10^2^ lower than that of the traditional SERS method.^[^ ^111^ ^]^ In another case, bacterial pathogens were immobilized on 3D plasmonic nanopillar arrays. The surfaces of bacteria were labeled with SERS tags, and further quantitative analysis was conducted for SERS detection of bacteria by collecting Raman mapping for statistical analysis.^[^ ^52^ ^]^ Usually, the more pixels, the higher the resolution. However, the higher the resolution, the more time needed. Therefore, it is necessary to determine the most suitable number of Raman mapping points to reduce the assay time. Pearson et al. established a SERS sandwich method with 3‐mercaptophenylboronic acid (3‐MPBA) as a capturer and signal to detect Enterococcus and monocytes. This method can detect common bacterial cells at concentrations as low as 100 CFU mL^−1^. Visual observation provides an effective method for the rapid screening of cells.^[^ ^112^ ^]^

### 4.2 Microfluidic System and Dielectrophoresis

**Figure 10:**
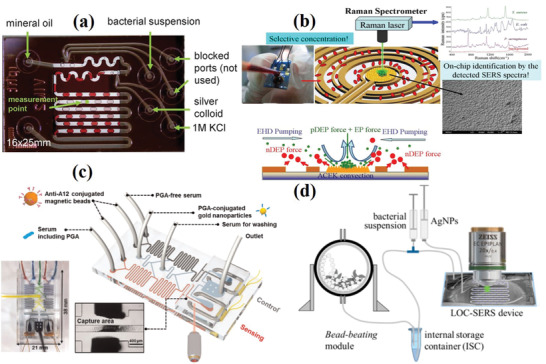
a) Applied microfluidic chip with 6 injection ports (4 used, 2 blocked). Reproduced with permission.^[^ ^113^ ^]^ Copyright 2011, The Royal Society of Chemistry. b) Illustration of the hybrid mechanism of selective concentration over a wide range asymmetric electrode array. Reproduced with permission.^[^ ^114^ ^]^ Copyright 2013, Elsevier. c) Scheme of the solenoid‐embedded dual channel microfluidic sensor for SERS‐based competitive immunoassay. Reproduced with permission.^[^ ^117^ ^]^ Copyright 2015, Elsevier. d) Scheme of a closed droplet based lab‐on‐a‐chip device for the differentiation of six species of mycobacteria. Reproduced with permission.^[^ ^116^ ^]^ Copyright 2016, American Chemical Society.

The main advantage of a microfluidic system is that it requires lower sample volumes. As a result, it has a high‐speed throughput compared with other technologies and has been gradually applied in the field of biological analysis in recent years. The lab‐on‐a‐chip can integrate various processes of sample preparation, separation, and detection. A lab‐on‐a‐chip device was designed for bacterial classification. Walter et al. reported that microfluidic devices have great potential for flow SERS detection of bacterial classification. Generally, the microfluidic equipment has 6 injection ports, 2 of which are not used. The four active injection ports are equipped with potassium chloride solution, mineral oil, silver colloid and bacterial suspension and then enter the chip. Among these, the main role of mineral oil is as a separation medium, and colloidal solution or aqueous analytes in mineral oil form segmented flow due to the formation of droplets as shown in **Figure** **10**a.^[^ ^113^ ^]^ Combined with dielectrophoresis (DEP), the rapid electrokinetic separation and concentration of bacteria from human blood on a microfluidic platform for on‐chip SERS detection and analysis has been developed (Figure 10b).^[^ ^114^ ^]^

The microfluidic platform was found to help SERS distinguish different foodborne pathogens.^[^ ^115^ ^]^ Muhlig et al. developed a closed droplet‐based lab‐on‐a‐chip device for the differentiation of six species of mycobacteria (Figure 10d).^[^ ^116^ ^]^ Similarly, Choi et al. developed an automatic SERS microdrop platform for the immunoassay of plague specific antigen fraction 1 (F1). A rapid and efficient immune response is achieved by continuous droplet generation, transport, and fusion. This integration provides a multi‐functional detection platform for multistep immunoassays in nL droplets. The LOD of F1 was 59.6 pg mL^−1^, and its detection sensitivity was 10^2^ for the traditional enzyme‐linked immunosorbent assay.^[^ ^85^ ^]^ The combination of microfluidics has the benefits of reduced sample consumption (usually µL or nL level), rapid assay times, and fully automated fluid control. An SERS‐based solenoid microfluidic sensor was reported for the quantitative detection of poly‐*γ*‐d‐glutamic acid (PGA), a biomarker of anthrax. The principle of this sensor is that the coupling reaction under microfluidic conditions is different, and there is competition in PGA and PGA‐conjugated Au NPs and anti‐PGA immobilized magnetic beads. The magnetic immune complex structure can be captured by solenoids in the device, and the SERS signals are directly detected and analyzed. The low detection limit of PGA is 100 pg mL^−1^ in serum (Figure 10c).^[^ ^117^ ^]^

### 4.3 Microarray Chip

**Figure 11:**
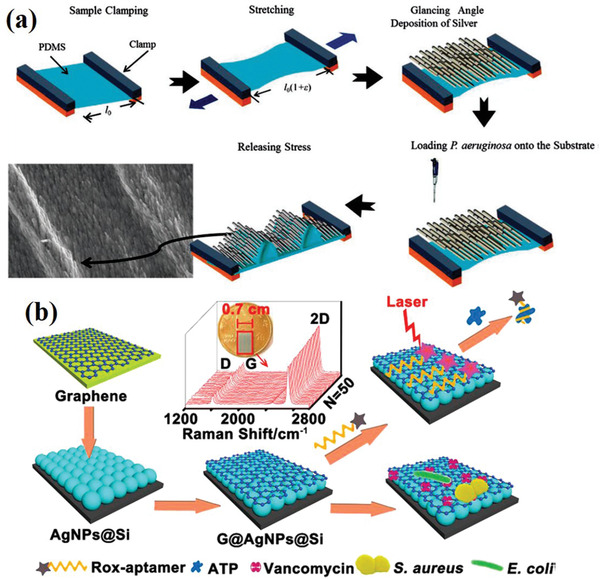
a) Schematic of buckled PDMS silver nanorod arrays as active 3D SERS cages for bacterial sensing. Reproduced with permission.^[^ ^43^ ^]^ Copyright 2015, The Royal Society of Chemistry. b) A graphene‐Ag NPs‐silicon SERS chip for quantitative detection of bacteria. Reproduced with permission.^[^ ^120^ ^]^ Copyright 2018, American Chemical Society.

In recent years, with continuous improvements in the performance of Raman microscopy system, Raman spectroscopy has been widely used in the field of microarray readout and bacterial detection. In short, microarray has become a promising technology for solving a variety of analytical problems in the field of biological analysis (**Figure** **11**a).^[^ ^43^, ^118^ ^]^ The microarray chip is a 2D array of specific analytical probes, which are deposited on a substrate (a glass sheet) in an addressable manner. Here, selective receptors found on the surface of substrate are exposed to the analyte.^[^ ^119^ ^]^ Immunoassays provide the possibility to selectively capture specific structures (e.g., bacteria, viruses, and small molecules). Using this method, high repeatability, specificity, and strong SERS spectra can be obtained from different kinds of bacteria fixed on the chip. Our team previously carried out the sensitive detection of single‐cell bacteria on a microarray platform.^[^ ^20^ ^]^ Raman‐based microarray readout technology has significantly improved the reproducibility of Raman analysis, mainly due to the combination of capture analyte and light on microarray, which ensures the stable interaction between the SERS active medium and analyte, making it especially suitable for the SERS analysis of small microbial molecules. He et al. developed a novel SERS chip, graphene(G)‐silver nanoparticles (Ag NPs)‐silicon (Si) (G@Ag NPs@Si) sandwich nanohybrids. This G@Ag NPs@Si platform can achieve molecular quantification and cellular analysis in real systems. Moreover, the chip is a multifunctional platform that can capture, discriminate, and inactivate bacteria simultaneously (Figure 11b).^[^ ^120^ ^]^

### 4.4 Data Processing Methods Combined with SERS Bacterial Detection

Data processing is a key step for the use of SERS in the detection of many kinds of bacteria. The main reasons are as follows: 1) Raman signals may be produced by many kinds of components of cells or tissues, and the spectrum produced is complex and disturbing; 2) the discrimination of different kinds of bacteria samples can only be based on the subtle difference in the Raman signal; 3) the basic components of different species of bacteria are very similar, resulting in similar SERS signals; 4) even the same bacterial species, different culture methods, and culture conditions may result in different SERS signals; 5) the Raman spectrum itself has some shortcomings such as signal instability. Therefore, data processing methods are necessary to obtain better results.

Principal component analysis (PCA) and cluster analysis are mainly used for the discrimination of several known pathogen species from each other. PCA is used to identify the variation in order to isolate different bacterial species accurately and reliably. For example, *Lactobacillus casei* and *Listeria monocytogenes* were successfully discriminated by applying them to their specific spectral data.^[^ ^121^ ^]^ Avci et al. analyzed the spectral changes of seven different UTI‐related bacteria at different growth stages, and the sources of spectral bands were described in detail. The results showed that regardless of the growth stage of the bacteria, all species could be distinguished using this PCA method.^[^ ^122^ ^]^ Isotopic labeling of ^13^C and/or ^15^N isotopes combined with SERS has been used for the differentiation and quantitative detection of phenotypically identical *E. coli* cells at the molecular level.^[^ ^123^ ^]^ This was achieved based on the redshifts of SERS spectral bands. The Raman shifts in the SERS spectral data were analyzed using partial least square regression (PLSR) and discriminant function analysis (DFA).

Chemometrics is another statistical method commonly used in bacterial SERS detection. A recent study reported on its use for the serotyping and detection of *Salmonella*, where stoichiometric analysis of the collected SERS spectra of *Salmonella* was conducted, as well as its precision of detection and characterization in unknown mixtures. In addition, the SERS detection and serum identification of other kinds of *Salmonella* have obtained an accuracy of detection and identification of 87–100%, and 67–100%, respectively. When the mixed samples are composed of six different bacteria at the same time, the accuracy of detection ranges from 65% to 100%, and the accuracy of detection is reduced for the simple mixed samples. In a word, this method can provide similar cheap substitutes over a long period of time; however, the reproducibility and accuracy of spectral detection still need to be further improved.^[^ ^124^ ^]^

Until now, although many methods have been established to identify unknown bacteria, the identification of bacteria in clinical microbiology is still a complex, time‐consuming and challenging exercise. To solve this problem, Cheong et al. proposed a multisite sequencing method for the rapid identification of clinical *Klebsiella pneumoniae*. The main idea was to identify three colonies of atcc70063 (control), st11, and St15 by SERS and multivariate statistical methods. The results showed that the Raman shift (high correlation) of the three colonies was similar to that of *E. coli*, but the corresponding Raman vibration modes were different.^[^ ^125^ ^]^

### 4.5 Nucleic Acid Amplification

**Figure 12:**
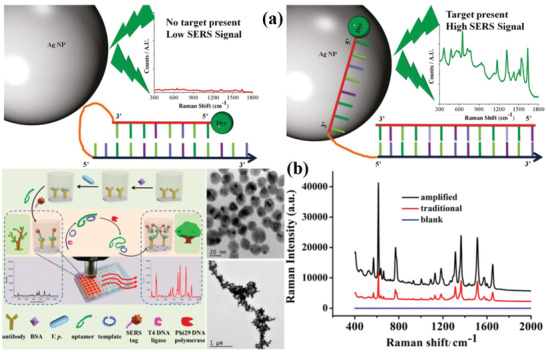
a) Schematic representation of a negative sample (no target present) resulting in a low SERS response, a positive sample resulting in a high SERS response. Reproduced with permission.^[^ ^126^ ^]^ Copyright 2011, American Chemical Society. b) Schematic and the detection results of in vitro isothermal nucleic acid amplification assisted SERS for the detection of *V. parahaemolyticus*. Reproduced with permission.^[^ ^128^ ^]^ Copyright 2017, American Chemical Society.

For the successful detection of pathogens, a more favored method is the detection and identification of specific pathogenic DNA sequences using SERS with a specifically designed SERS primer. When the sample is negative (the target DNA does not exist), the dye‐labeled SERS primers are partially closed, and the main component is dsDNA. With a positive sample, the complementary target DNA will replace some of the complementary segments of the SERS primer. The dye‐labeled SERS primers will partially turn into ssDNA and interact with the surface of NPs to enhance the SERS response. This strategy has been applied to the DNA detection of various pathogens. The combination of PCR and SERS was used to effectively detect the specific DNA segments by van Lierop's group (**Figure** **12**a).^[^ ^126^, ^127^ ^]^

Nucleic acids amplification can be used for the assembly of metallic nanoparticles. In one study, SERS technology and in vitro constant temperature amplification were combined to detect *V. parahaemolyticus* with high sensitivity. The main steps are as follows: first, the antibody is fixed on the microplate, in the presence of the target bacteria, and the aptamer of ssDNA is added to form the sandwich structure of the antibody‐target aptamer. Using Au@Ag NPs as SERS substrate, the stretched ssDNA products were labeled and detected by SERS after isothermal rolling circular amplification (RCA). The RCA process is conducive to the assembly of Au@Ag NPs and provides a large space for the formation of high‐density Raman “hot spots.” Using this enhanced Raman signal strategy, the LOD of the bacteria can be as low as to 1 CFU mL^−1^ successfully for the detection of *V. parahaemolyticus* (Figure 12b).^[^ ^128^ ^]^ After further optimization, a new detection method was established, and three pathogens of bacterial meningitis, including two complementary DNA probes, were hybridized to the target sequence at the same time, and digested the double‐stranded DNA l‐exonuclease. The digested products were examined by SERS measurement.^[^ ^129^ ^]^ The quantitative detection of three meningitis pathogens in multiple experiments has been realized successfully. The detection limit calculated is in the range of picomolar, which overcomes the problem of culture‐based methods. Liu et al. established a multiple diagnosis and detection platform for pathogen based on the synergistic effect of SERS technology and recombinase polymerase amplification (RPA). The SERS‐RPA platform detection method is faster and more sensitive than traditional PCR.^[^ ^130^ ^]^

### 4.6 Point‐of‐Care Testing

**Figure 13:**
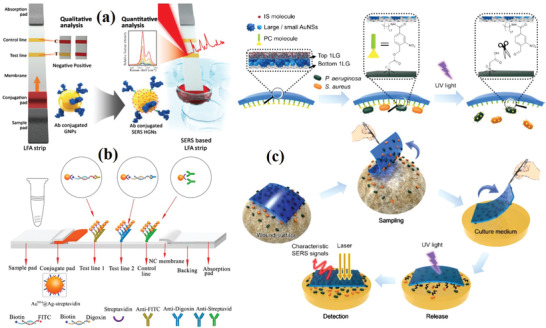
a) Schematic of conventional and the SERS‐based LFA strip. Reproduced with permission.^[^ ^132^ ^]^ Copyright 2016, The Royal Society of Chemistry. b) Schematic of the SERS‐based lateral flow strip biosensor for simultaneous detection of *L. monocytogenes* and *S. enterica*. Reproduced with permission.^[^ ^133^ ^]^ Copyright 2017, American Chemical Society. c) Schematic of an intelligent “three‐in‐one” adhesive tape for photo‐controlled release, rapid sampling, and SERS detection of pathogens from infected wounds. Reproduced with permission.^[^ ^134^ ^]^ Copyright 2019, American Chemical Society.

In order to meet the needs of rapid testing for bacterial detection in food safety and clinical diagnosis, point‐of‐care testing (POCT) has become an important trend in the detection of bacterial biomarkers.^[^ ^131^ ^]^ POCT is performed at the sampling site and uses portable analytical instruments and supporting reagents to obtain test results quickly. Lateral flow assay (LFA) is a common method of POCT. Conventional LFA strips using Au NPs as indicators have limits in quantitative analysis and low sensitivity, which can be overcome by fabricating SERS‐based LFA strips. Raman reporter‐labeled nanoparticles are used as SERS detection probes instead of Au NPs. The LFA band based on SERS can identify the presence of the target antigen by detecting the color changes in the region. In addition, based on the SERS signal in the test area, highly sensitive quantitative detection and evaluation can be carried out (**Figure** **13**a). Using this strategy, *Staphylococcal enterotoxin B* was detected with an LOD of 1 pg mL^−1^, and the sensitivity was ≈10^3^ more than that of the corresponding ELISA method.^[^ ^132^ ^]^ Based on SERS, the LFA strip combined with recombinase polymerase amplification (RPA) can be used for the simultaneous detection of monocytes and *S. enteritidis* (Figure 13b).^[^ ^133^ ^]^ In order to detect two pathogens at the same time, two test lines are needed. The capture antibodies (McAb FITC and McAb digoxin) were distributed on the detection line (line 1 and line 2) of NC membrane, while the control antibodies (McAb streptavidin) were distributed on the control line. By measuring the characteristic peak intensity of SERS, the highly sensitive quantitative detection of *S. enteritidis* and *S. monocytogenes* was realized. The detection limits were 27 and 19 CFU mL^−1^, respectively. Ju et al. reported an intelligent “three‐in‐one” adhesive tape for photocontrolled release, rapid sampling, and SERS detection of pathogens from infected wounds. This “three‐in‐one” tape was used to detect the concurrent infection of *P. aeruginosa* and *S. aureus* by pasting the adhesive tape on a skin burn wound. The analytical time only requires several hours; thus, this adhesive tape has great potential as a POCT device in field of health care (Figure 13c).^[^ ^134^ ^]^

## 5 Application of Bacterial Detection in Clinical and Food Safety

**Figure 14:**
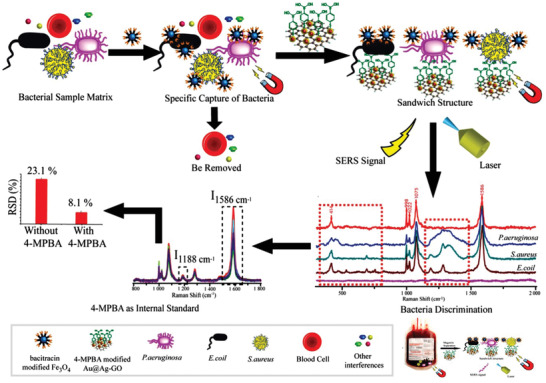
Schematic of bacterial detection via a SERS sandwich strategy, in which bacitracin modified magnetic Fe_3_O_4_ NPs were used to capture bacteria and 4‐MPBA modified Au@Ag‐GO nanocomposites were used as SERS tags. Reproduced with permission.^[^ ^146^ ^]^ Copyright 2018, The Royal Society of Chemistry.

The fingerprint identification characteristics of SERS technology has allowed it to become an important and effective method for distinguishing closely related bacteria growing on the same solid or liquid medium.^[^ ^135^ ^]^ The application of SERS for clinical bacterial detection and identification in human body fluids has gained much attention.^[^ ^136^, ^137^ ^]^ In recent years, there have been a series of reports about the clinical bacterial detection and identification of SERS. For example, Avci et al. analyzed and identified the pathogenic bacteria of urinary tract infection that had been cultured for 1 h by combining SERS and PCA.^[^ ^122^ ^]^ Tien et al. rapidly identified bacterial pathogens in the dialyzate of peritoneal dialysis peritonitis patients using SERS technique.^[^ ^138^ ^]^ Crawford et al. achieved the low‐level detection of a major virulence factor in the infectious pathology of *Mycobacterium tuberculosis*.^[^ ^139^ ^]^ Premasiri et al. reported and analyzed the SERS spectrum and detection results of 12 isolates of urinary tract infection (UTI) in clinical samples.^[^ ^140^ ^]^ Cheong et al. reported that the spectrum method based on SERS can be used to analyze the feasibility of unknown *K. pneumoniae* resistant strains in clinical laboratory. In addition, seven different UTI‐related or similar bacteria were used to analyze the spectral changes during the growth stages.^[^ ^125^ ^]^ SERS was also applied to characterize the bacterial biological activity and interaction of small molecules with bacteria. By investigating the changes of SERS spectra intensity when a susceptible strain is exposed to an antibiotic, the antibiotic‐induced chemical changes in bacterial cells can be analyzed.^[^ ^141^, ^142^, ^143^ ^]^ The main feature of Raman spectroscopy is that it can easily provide detailed fingerprint information of various molecules in the biological system. Therefore, it has a high potential for broad applications prospect in the study of the interaction between nanoparticles and microorganisms. For example, the effect of Ag NPs that are adsorbed on the surface of purple membranes of *Halobacterium salinarium* bacteria have been investigated using SERS.^[^ ^144^ ^]^ In order to better understand the biofilm formation and detection potential, SERS has been used to monitor the formation of biofilm by important microorganisms in clinical, such as *Candida albicans, P. aeruginosa*, and *S. epidermidis*.^[^ ^145^ ^]^ Our group developed a new sandwich structure biosensor for the isolation and detection of multiple bacterial pathogens (e.g., *S. aureus, E. coli*, and *P. aeruginosa*) via SERS tags and magnetic separation, where the lowest concentration was 101 CFU mL^−1^. Importantly, when this novel method was applied to detect bacteria from clinical patients, the validation analysis showed that the correct resolution of clinical blood samples was 97.3%. The multifunctional biosensor integrates the separation, detection and sterilization of bacteria, and has great prospect in clinical diagnosis and safe blood transfusion (**Figure** **14**).^[^ ^146^ ^]^

**Figure 15:**
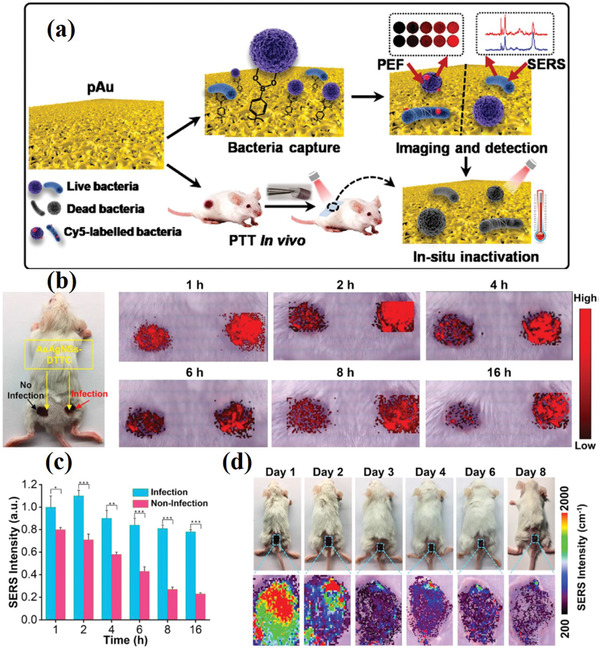
a) Schematic of the multifunctional chip for bacterial capture, imaging, detection, and in situ inactivation of pathogen bacteria in vivo. Reproduced with permission.^[^ ^147^ ^]^ Copyright 2020, The Royal Society of Chemistry. b) The images of uninfected and infected wounds at different times after the use of Au‐Ag NSs‐DTTC. c) The relationship between the SERS intensity and time of uninfected and infected wounds. d) The results and SERS images of wounds treated with Au–Ag NSs–DTTC at different time (1–8 d). Reproduced with permission.^[^ ^148^ ^]^ Copyright 2020, Elsevier.

Liu et al. constructed a multifunctional plasmonic Au chip for use in bacterial imaging and detection by double mode. The detection limit was as low as 102 CFU mL^−1^, and the results show good reproducibility and accuracy. The chip can distinguish Gram‐positive and Gram‐negative bacteria, which provides a very important reference value for clinical diagnosis and treatment. Moreover, the chip has excellent photothermal antibacterial activity, with a bactericidal rate up to 98%. It also inactivates Gram‐positive bacteria and Gram‐negative bacteria in situ, which is of great significance in the field of human health examination and early clinical diagnosis (**Figure** **15**a).^[^ ^147^ ^]^ Zhou et al. reported a new Raman tag 3,3’‐diethylthiatricarbocyanine iodide (DTTC)‐conjugated gold–silver nanoshells (Au–Ag NSs) substrate that provided a non‐invasive and extremely high sensitive detection (down to 300 CFU mL^−1^). The photothermal therapy of Au–Ag NSs gel is synergistic with silver release and is almost non‐toxic. The results show that Au–Ag NSs‐DTTC is an important and promising clinical antibacterial technology (Figure 15b–d).^[^ ^148^ ^]^

Pearson et al. compared the traditional aerobic plate counting (APC) and SERS sandwich methods based on the test results of the actual environment and food. The detection results of bacteria in pool water and spinach leaves showed that the SERS method could detect bacteria in fixed phase at a high level, with a sensitivity that was much higher than that of the APC method. However, the APC method has more advantages in terms of cell viability.^[^ ^149^ ^]^ Zheng et al. synthesized a multifunctional gold nanobones (GNBs) based on gold nanorods (GNRs) as SERS enhanced substrates, which were modified by aptamer‐1 (Apt‐1) and used to detect *E. coli* O157: H7 by signal molecule rhodamine B (RhB). Among them, Apt‐1 and RhB are uniformly embedded in the GNBs structure, and the detection results show that the SERS signal has high selectivity, high stability, and high sensitivity. In a word, this kind of highly sensitive and efficient sensor for the detection of *E. coli* O157: H7 can be further applied to the detection of pathogenic bacteria in food safety, and has broad application prospects in coming years.^[^ ^150^ ^]^

## 6 Summary and Further Perspectives

In this review, recent developments in SERS technology for the rapid, sensitive, and stable detection of bacteria are summarized. In short, owing to its high sensitivity, high flexibility, and in situ remote sensing ability, SERS technology has become a promising recognition and detection platform for the practical application of label‐based and label‐free detection in recent years.

In the future, the application of SERS technology in bacterial detection still needs to be improved in the following aspects: 1) fabricate highly active SERS substrates with excellent uniformity to dramatically enhance the weak Raman signal of low concentration microorganisms; 2) improve the ability of SERS‐active substrates to increase the interaction or adsorption capacity between biological samples and bacteria. Novel recognition elements for bacteria can be used to improve capture and cut costs. Antibacterial peptides and aptamers are becoming potentially promising alternatives to antibodies; 3) improve the capability of enrichment and concentration of target bacteria from low‐content liquid samples; 4) for the label‐based SERS method, it is necessary to improve the stability of SERS tags in order to obtain higher reproducibility of Raman signals. Background‐free Raman reporters, such as alkyne (C≡C) and nitrile (C≡N), can be used as reporters to eliminate spectral mixing and background interference; 5) develop novel and ingenious methods to precisely achieve the band assignment of the SERS spectra of bacteria; most of the currently reported cases and literatures are only tentative in assignment, and the exact substances on the bacterial cell wall of their SERS signals are still unknown.

Last but not least, with continuous improvements in high sensitivity, multichannel, multifunction, simple, and cost‐effective instrument performance, the application prospects of SERS technology in POCT diagnosis are very broad. Therefore, it is time to shift the SERS‐based bacterial detection from laboratory research and the development stage into clinical and POCT area. In the next few years, SERS will be used as a rapid pathogen diagnostic tool. To achieve this goal, several efforts have to be made: 1) establish a good method for the elimination of interference in the complex matrices of “real world’’ samples, such as blood and urine; 2) shorten the time between sample collection and final diagnostic result; 3) develop SERS‐combined systems that are small, rugged, and easy to use while still being cost effective; 4) according to fine chemical fingerprint spectrum information provided by Raman spectrum, some miniaturized and novel biomedical bacterial detection instruments based on microfluidic chips for clinical POCT analysis could be developed and manufactured.

## Conflict of Interest

The authors declare no conflict of interest.
